# Weight Loss Outcomes following Roux-en-Y Gastric Bypass and Sleeve Gastrectomy in an Ethnically Diverse Bariatric Population: Which Is More Effective?

**DOI:** 10.1155/2021/9702976

**Published:** 2021-04-16

**Authors:** Saqib Saeed, Leaque Ahmed, Khuram Khan, Sanjiv Gray, Kashif Saeed, Hector DePaz, Amrita Persaud, Bianca Passos-Fox, Kevin C. J. Zhang, Sara Alothman, Paritosh Suman

**Affiliations:** ^1^Department of Surgery, Montefiore Medical Center, Bronx, NY, USA; ^2^Department of Surgery, Harlem Hospital Center, New York, NY, USA; ^3^Department of Surgery, Wyckoff Heights Medical Center, Brooklyn, NY, USA; ^4^Department of Surgery, Osceola Regional Medical Center, Kissimmee, FL, USA; ^5^Department of Surgery, Cleveland Clinic Florida, Weston, FL, USA; ^6^University of California, Berkeley, CA, USA

## Abstract

**Background:**

Laparoscopic Roux-en-Y gastric bypass (LRYGB) and laparoscopic sleeve gastrectomy (LSG) have comparable weight loss outcomes in a general bariatric population.

**Objectives:**

This study aimed to investigate whether similar outcomes can be observed in Hispanic and African American population. *Settings*. Community Hospital in New York, New York, United States.

**Methods:**

The 5-year prospective data of patients who underwent LRYGB and LSG at a single center were retrospectively reviewed. The long-term weight loss outcomes between patients who had LRYGB and LSG were compared after adjusting for age, sex, race, diabetes mellitus, and hypertension with the linear mixed-effects or logistic regression model.

**Results:**

Most patients were Hispanic (59.2%) and African American (22.7%). The mean% total weight loss (%TWL) values of patients with BMI <45 kg/m^2^ who underwent LRYGB and LSG were 73% and 62% after 1 year, 69% and 56% after 2 years, and 71% and 54% after 5 years, respectively. In patients with a BMI of 45–50 kg/m^2^ who underwent LRYGB and LSG, the mean %TWL values were 69% and 56% after 1 year, 75% and 58% after 2 years, and 57% and 45% after 5 years, respectively. Meanwhile, the %TWL values of patients with BMI >50 kg/m^2^ who had LRYGB and LSG were 53% and 42% after 1 year, 53% and 45% after 2 years, and 49% and 36% after 5 years, respectively. All results were statistically significant (*p* < 0.0001) and remained valid after adjusting for cofactors.

**Conclusion:**

Thus, LRYGB had consistent and sustained long-term weight loss outcomes compared with LSG in a predominantly ethnically diverse patient population with different BMI. Our study had several limitations in that it is retrospective in nature and some patients were lost to follow-up during the study period.

## 1. Introduction

The prevalence of obesity and its related diseases is increasing in the US and other countries worldwide. Obesity in adults is defined as a body mass index (BMI) greater ≥30 kg/m 2. According to the Centers for Disease Control and Prevention, the prevalence rate of obesity is 39.8%, and 93.3 million adults present with such condition in the US [1]. The annual cost for the medical management of obesity in the US was approximately $147 billion in 2008. In 2016, 1.9 billion adults were overweight and 650 million had obesity worldwide [2]. The prevalence of such condition worldwide has tripled from 1975 to 2016 and is higher in African Americans and Hispanic adults. Compared with underweight, obesity has been associated with a high number of deaths worldwide [2]. The prevalence of the disease may increase to 41% in men and 78% in women by 2022 [3]. In addition, its prevalence in adolescents has increased, thereby causing an early onset of comorbidities, such as hypertension (HTN), dyslipidemia, type 2 diabetes mellitus (T2D), and obstructive sleep apnea [4].

Bariatric surgery is the most effective treatment option for obesity, which results in a substantial and sustained weight loss with improvement and often resolution of obesity-related comorbidities. Laparoscopic Roux-en-Y gastric bypass (LRYGB) and laparoscopic sleeve gastrectomy (LSG) are the most commonly performed bariatric procedures worldwide, and the use of LSG is still increasing [5]. LSG is technically less challenging, has a shorter operative time, and has fewer complications than LRYGB. Sleeve gastrectomy (LSG) has been compared with gastric bypass in several randomized trials. However, the study results were contrasting in terms of %TWL, BMI, and comorbidities [6]. Prior studies have indicated that weight loss after bariatric surgery may be less pronounced in African Americans [7] and Hispanics than in non-Hispanic whites [8]. Thus, the current study aimed to compare the efficacy of LSG and LRYGB in a predominantly Hispanic and African American population with morbid obesity.

## 2. Materials and Methods

The electronic medical records of patients (*n* = 2631) who had undergone LRYGB or LSG at a single institution between 2004 and 2018 were reviewed. The research protocol was approved by our institutional review board. Demographic and clinical data were collected retrospectively during preoperative visits; at 1, 3, 6, and 9 months after surgery; and annually thereafter up to 5 years. The demographic data included age, gender, and race/ethnicity, and the clinical data were date/type of bariatric surgery, height (cm), weight (kg), presence/absence of T2D, and HTN. T2D was defined as the intake of oral hypoglycemic agents or insulin or fasting plasma glucose level of at least 126 mg/dL (6.9 mmol/L). Meanwhile, HTN was defined as a systolic blood pressure ≥140 mmHg or a diastolic blood pressure ≥90 mmHg measured on two separate occasions or the use of antihypertensive agents.

### 2.1. Surgical Procedure

All participants underwent either LRYGB or LSG. The length of the Roux limb in patients who underwent LRYGB was 150 cm. The Roux limb was brought up in an antecolic, antegastric manner. Postoperatively, all patients were managed in a monitoring unit and were then transferred to the bariatric department on postoperative day 1 after completing an upper gastrointestinal study. All patients who were on our standard bariatric liquid diet on postoperative day 2 were discharged to home and provided with instructions to continue the diet for 1 week. The patients were evaluated in the clinic at 1 week and 1 month after surgery and every 3 months within the first year, then every 6 months at 2 years, and then, annually.

### 2.2. Statistical Analysis

All statistical analysis were conducted using SAS version 9.1.4 (SAS Institute, Cary, NC). Multiple measurements were recorded during the study period for each participant. Therefore, to assess the correlation between observations in each participant, a linear mixed-effects model analysis was conducted for each continuous outcome variable. Meanwhile, for each categorical outcome variable, a logistic regression analysis for repeated measures was performed to assess the relationship between the outcome variable and predictors of interest, including time, age, sex, race, surgical procedure, diabetes, and HTN. In all analyzes, a *p* value <0.05 was considered statistically significant.

## 3. Results

The patients were divided into three groups according to their preoperative BMI. Group 1 comprised patients who had BMI <45 kg/m^2^; group 2, patients with a BMI of 45–50 kg/m^2^; and group 3, patients with BMI >50 kg/m^2^. The demographic data of each group are shown in Table 1.

Most patients (59%) were Hispanics, and 22% were African Americans. LSG was performed in 62% of the patients and LRYGB in 38%. Approximately 41.0% and 26% of the patients presented with HTN and T2D. The mean BMI of patients who underwent LRYGB was 44 kg/m^2^, and that of patients who had LSG was 45.5 kg/m^2^(*p* > 0.05). The excess weight at baseline was significantly higher in men than in women (mean difference = 29.20, *p* < 0.0001) and in African Americans than in to Hispanics (mean difference = 13.56, *p* < 0.0001).

**Figure 1:**
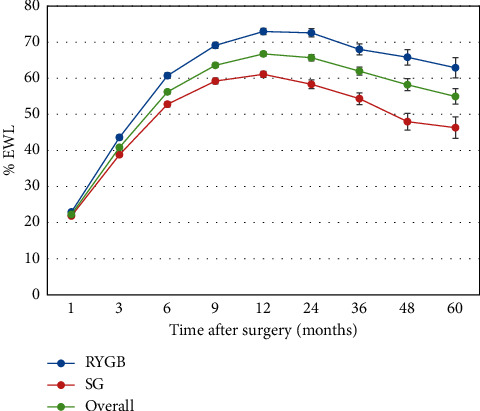
TWL (mean, SE) after surgery during the 5-year study period.

The %TWL in patients with BMI <45 kg/m^2^ was higher than that in patients with BMI >45 kg/m^2^(*p* < 0.0001) during the 5-year study period. The mean %TWL of the patients who underwent the two procedures is shown in Figure 1.

### 3.1. Percent Excess Weight Loss in Patients with BMI <45 kg/m^2^

**Figure 2:**
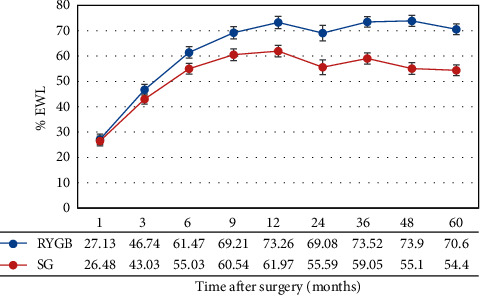
TWL (mean, SE) during the 5-year study period in group 1 (BMI <45 kg/m^2^).

In terms of %TWL, no significant difference was observed between patients who underwent LRYGB and LSG at 1 and 3 months after surgery (*p*=1.00 and 0.09, respectively). However, a significant difference (*p* < 0.0001) was observed in %TWL at 6 and 9 months (mean difference = 6.44 and 8.67, respectively) and at 1, 2, 3, 4, and 5 years after surgery (mean difference = 11.29, 13.49, 14.47, 18.8, and 16.2, respectively), as shown in Figure 2. The %TWL did not significantly differ in terms of gender and race, as shown in Table 2. Hypertensive and nondiabetic patients had significantly higher excess weight loss than nonhypertensives (%TWL of 4%–10% at different intervals, *p* < 0.0001).

### 3.2. Percent Excess Weight Loss in Patients with a BMI of 45–50 kg/m^2^

**Figure 3:**
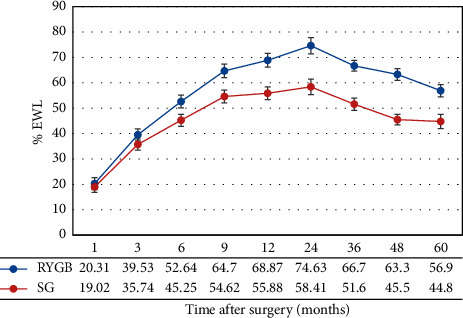
TWL (mean, SE) during the 5-year study period in group 2 (BMI of 45–50 kg/m^2^).

In the group with a BMI of 45–50 kg/m^2^, a significant difference (*p* < 0.0001) was observed in %TWL between patients who underwent LRYGB and LSG at 6 and 9 months (mean difference = 7.39 and 10.08, respectively) and at 1, 2, 3, 4, and 5 years after surgery (mean difference = 12.99, 16.23, 15.1, 18.8, and 5 12.1, respectively), as shown in Figure 3. In African Americans and Hispanics, the mean difference in %TWL was −2.85 (*p*=0.0320). No significant difference was observed in terms of age and presence of T2D or HTN.

### 3.3. Percent Excess Weight Loss in Patients with a BMI >50 kg/m^2^

**Figure 4:**
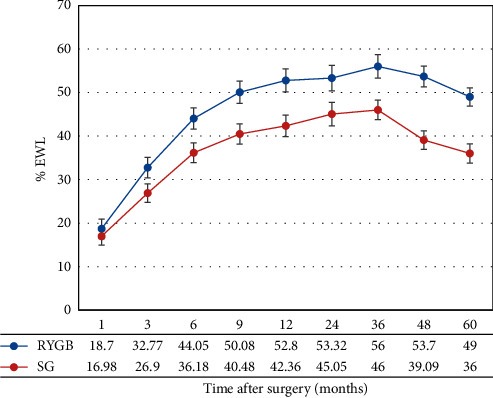
TWL (mean, SE) during the 5-year study period in group 3 (BMI ≥50 kg/m^2^).

A significant difference (*p* < 0.001) was observed in %TWL between patients who underwent LRYGB and LSG at 3, 6, 9, and 12 months (mean difference = 5.87, 7.87, 9.59, and 10.43, respectively) and at 2, 3, 4, and 5 years after surgery (mean difference = 8.27, 10, 14.61, and 9, respectively), as shown in Figure 4. Nondiabetic patients had a higher %TWL than diabetic patients (39.64 vs. 36.10, *p*=0.03). Age, sex, race, and HTN did not have any significant effect on %TWL.

## 4. Discussion

Morbid obesity is a modern epidemic, and its prevalence among adults, adolescents, and, recently, children has been increasing in the US and other countries worldwide [9]. Bariatric surgery has consistent outcomes. That is, it results in significant and sustained weight loss. Both LRYGB and LSG are well established weight loss procedures. Initially, LRYGB was considered the procedure of choice for long-term sustained weight loss in extremely obese patients with comorbidities. Recently, LSG has surpassed gastric bypass as the most common procedure owing to its comparable weight loss outcomes, as shown in different studies.

In a meta-analysis and systemic review of nine randomized clinical trials, Osland et al. have shown nonsignificant weight loss outcome differences between LRYGB and LSG [10]. The Sleeve vs. Bypass (SLEEVEPASS) trial and the Swiss Multicenter Bypass or Sleeve Study (SMBOSS), which are two randomized clinical trials conducted recently, have shown the weight loss outcomes of both gastric bypass and LSG. The SLEEVEPASS enrolled 240 patients with 1 : 1 randomization. In this study, the LRYGB and LSG groups achieved a %TWL of 57% and 49% after 5 years, respectively, and no statistically significant difference was observed between the two groups [11]. Meanwhile, the SMBOSS included 217 patients who were randomized 160 either to gastric bypass or LSG. The trial did not show a statistically significant difference in %TWL between patients who underwent the two procedures. The LRYGB and LSG groups achieved a %TWL of 68.3% and 61.1%, respectively, after 5 years [12].

Shoar and Saber included 14 studies in his meta-analysis comparing the midterm and long-term weight loss outcomes between LRYGB and LSG. Both procedures did not significantly differ in terms of midterm weight loss outcomes (3–5 years). However, the long-term weight loss outcomes (>5 years) of LRYGB were significantly superior to those of LSG [13]. Similarly, Li et al. have included 62 studies in their meta-analysis favoring LRYGB over LSG for sustained excess weight loss [14]. Ignat et al. have reported that gastric bypass had a higher %TWL than LSG in their randomized clinical trial. In total, 45 patients were randomized to LRYGB and 55 to LSG. The patients who underwent LRYGB and LSG had a %TWL of 74.8% and 65.1%, respectively, after 5 years [15]. To date, the largest study included 47,101 patients from three national registries (Sweden, Norway, and the Netherlands). The authors reported that patients who had LRYGB had a higher total weight loss than those who underwent LSG (95.8% vs. 84.6%) after 1 year [16].

Most of the aforementioned studies have analyzed the Caucasian bariatric population. Bariatric outcomes in ethnic minorities (African Americans and Hispanics) have not been assessed in large-scale studies. Previous studies have indicated that ethnic minorities have a lower %TWL than Caucasians [17]. Madan et al. assessed the weight loss outcomes in 30 African American and 67 Caucasian patients after LRYGB. Results showed that the Caucasian patients had a higher %TWL than African Americans (74% vs. 66%). However, no significant difference was observed in achieving successful weight loss [18]. Buffington and Marema reported that Caucasians and AA had a %TWL of 80% and 63% after LRYGB at 12–18 months after surgery [19].

Elli et al. assessed the bariatric outcomes in ethnic minorities and showed that African American patients had a lower %TWL than non-Hispanic white and Hispanic patients after 6 months. No significant differences were observed in %TWL between Hispanics and whites. Moreover, patients who had LRYGB had a higher %TWL than those who underwent LSG [20].

Serano et al. reported that the surgical procedures had a lower efficacy in Hispanic populations. However, the %TWL of patients who underwent LRYGB was higher than that of patients who had either LSG or laparoscopic gastric band in these populations [21].

Our demographic data are consistent with those of prior studies, which included mostly women and participants aged younger than 55 years. The mean preoperative BMI of 45.24 kg/m^2^ in our study is similar to that reported in the Bariatric Outcomes Longitudinal Database published in 2010 [22]. In this study, most populations comprised Hispanics and African Americans. At baseline, the African Americans had a higher weight than Hispanics (114.63 vs. 101.06 kg, *p* < 0.001). The %TWL of African Americans was significantly lower than that of Hispanics at 3 months to 2 years after surgery (mean difference = 4%–8% during a different study period, *p* < 0.0001), and this finding is similar to that of prior studies. When %TWL was assessed based on BMI, no significant difference was observed between AA and Hispanic patients with BMI <45 and >50 kg/m^2^. In the group with a BMI of 45–50 kg/m^2^, the AA had a significantly lower %TWL at different point intervals than Hispanics.

In our study, patients who underwent LRYGB had significantly higher %TWL than those who had LSG in all three BMI groups. The current study that predominantly included Hispanics and African American patients adds to the growing literature that highlights the efficacy of bariatric surgery for sustained long-term weight loss. Bariatric surgery can be considered by healthcare professionals as a cost-effective tool in managing obesity epidemic and its related comorbidities, particularly among minorities.

Our study had several limitations in that it is retrospective in nature and some patients were lost to follow-up during the study period (Table 3). Moreover, only few male patients were included, and the population comprised only ethnic minorities.

## 5. Conclusions

LRYGB results in consistent and sustained long-term weight loss in a predominantly Hispanic and African American patient population with different BMIs, and individuals who undergo LRYGB have a higher %TWL than those who had LSG.

## Figures and Tables

**Table 1:** Demographic and baseline characteristics of the participants.

Demographic characteristics of the participants (*N* = 2631)	Overall	Group 1 (patients with BMI <45 kg/m^2^), *n* = 1492	Group 2 (patients with BMI 45–50 kg/m^2^), *n* = 547	Group 3(patients with BMI ≥45 kg/m^2^), *n* = 592
Age (years)	37 (18–76)	41 (18–70)	39 (18–74)	39 (18–72)

BMI (kg/m^2^)	45 ± 7	40.3 ± 2.8	47.4 ± 1.4	50.4 ± 5.9

Sex				
Female	2332 (88.64%)	1364 (91.4%)	489 (89.3%)	477 (80.5%)
Male	299 (11.36%)	128 (8.6%)	58 (10.7%)	115 (19.5%)

Race/ethnicity				
Hispanic	1559 (59.28%)	979 (65.6%)	317 (57.9%)	259 (43.7%)
African American	598 (22.75%)	252 (16.8%)	139 (25.4%)	208 (35.1%)
Others	474 (17.97%)	261 (17.4%)	91 (16.6%)	125 (21.1%)

Surgical procedure (*n*, %)				
LSG	1618 (61.5%)	915 (61.3%)	314(57.4%)	389 (65.7%)
LRYGB	1013 (38.5%)	577 (38.7%)	233 (42.6%)	203 (34.3%)

Comorbidities (*n*, %)				
Diabetes	675 (25.69%)	383 (25.67%)	133 (24.31%)	160 (27.02%)
Hypertension	1066 (40.69)	586 (39.27%)	213 (38.93)	277 (46.79%)

BMI: body mass index, LSG: laparoscopic sleeve gastrectomy, LRYGB: laparoscopic Roux-en-Y gastric bypass, kg: kilogram, m: meter.

**Table 2:** Results of the mixed-effects model (type III F-test, outcome = % of excess weight loss).

Group 1				Group 2				Group 3			
Effect	Num DF	Den DF	F-test	*p* value	Num DF	Den DF	F-test	*p* value	Num DF	Den DF	F-test	*p* value
Procedure	2	1422	41.78	<0.0001	2	520	40.60	<0.0001	2	536	28.37	<0.0001
Age	1	1422	9.46	0.0021	1	520	0.40	0.5249	1	536	4.12	0.0428
Sex	1	1422	0.38	0.5363	1	520	22.40	<0.0001	1	536	3.44	0.0643
Race	2	1422	3.09	0.0559	2	520	3.21	0.0410	2	536	2.65	0.0716
Diabetes	1	1422	21.22	<0.0001	1	520	2.75	0.0979	1	536	4.69	0.0308
Hypertension	1	1422	9.47	0.0021	1	520	1.58	0.2094	1	536	6.43	0.0115
Time after surgery	5	3663	46.09	<0.0001	5	1315	37.82	<0.0001	5	1455	22.88	<0.0001

Num DF: number of degrees of freedom in the model, Den DF: number of degrees of freedom associated with the model errors.

**Table 3:** Results with various post-BMI and lost to follow-up.

	BMI< 45, *n* = 1492	Lost to follow-up	BMI 45–50, *n* = 547	Lost to follow-up	>50, *n* = 592	Lost to follow-up
Postop 1 month	1355	137	508	39	518	74
Postop 1 year	803	689	290	257	324	268
Postop 2 year	428	1064	180	367	193	399
Postop 3 year	267	1225	128	419	114	478
Postop 4 year	130	1362	74	473	78	514
Postop 5 year	80	1412	36	511	43	549

Postop: postoperation follow-up, BMI: body mass index.

## Data Availability

The data that support the findings of this study are available from the corresponding author (SS) upon reasonable request.

## Abbreviations

BMI: Body mass index
T2D: Type 2 diabetes mellitus
%TWL: Percent total weight loss
HTN: Hypertension
LRYGB: Laparoscopic Roux-en-Y gastric bypass
LSG: Laparoscopic sleeve gastrectomy
